# Noncovalent Bonds through Sigma and Pi-Hole Located on the Same Molecule. Guiding Principles and Comparisons

**DOI:** 10.3390/molecules26061740

**Published:** 2021-03-20

**Authors:** Wiktor Zierkiewicz, Mariusz Michalczyk, Steve Scheiner

**Affiliations:** 1 Faculty of Chemistry, Wrocław University of Science and Technology, Wybrzeże Wyspiańskiego 27, 50-370 Wrocław, Poland; 2 Department of Chemistry and Biochemistry, Utah State University Logan, Logan, UT 84322-0300, USA; steve.scheiner@usu.edu

**Keywords:** molecular electrostatic potential, halogen bond, pnicogen bond, tetrel bond, chalcogen bond, cooperativity

## Abstract

Over the last years, scientific interest in noncovalent interactions based on the presence of electron-depleted regions called σ-holes or π-holes has markedly accelerated. Their high directionality and strength, comparable to hydrogen bonds, has been documented in many fields of modern chemistry. The current review gathers and digests recent results concerning these bonds, with a focus on those systems where both σ and π-holes are present on the same molecule. The underlying principles guiding the bonding in both sorts of interactions are discussed, and the trends that emerge from recent work offer a guide as to how one might design systems that allow multiple noncovalent bonds to occur simultaneously, or that prefer one bond type over another.

## 1. Introduction

The concept of the σ-hole, introduced to a wide audience in 2005 at a conference in Prague by Tim Clark [1], influenced a way of thinking about noncovalent interactions that prevails to this day. Early experimental findings [2,3,4,5] of unusual halogen·halogen contacts were explained in part by the anisotropic distribution of electronic density around a halogen atom when linked to an electron-withdrawing group. Already in 1992, it had been learned that electronegative atoms from Groups 14–17 have regions of positive molecular electrostatic potential (MEP) on their outer surfaces, along an extension of a covalent bond, which may attract an incoming Lewis base [6]. This observation led to the further computational studies which resulted in formulation of the σ-hole idea [7] which was further generalized in later works [7,8,9,10,11,12,13,14,15,16,17]. These ideas concerning the halogen bond were successfully adapted to atoms from other families of the periodic table, which were later grouped into the category of σ-hole bonds. This general sort of noncovalent bond has been subdivided by the specific family from which the bridging atom is drawn, i.e., chalcogen [18,19,20,21,22,23], pnicogen (pnictogen) [24,25,26,27,28], or tetrel bonds [29,30,31]. The former, along with the halogen bond, has been formally recognized and detailed in recent IUPAC recommendations [32,33].

Quite the opposite from representing exotic or unusual contacts, these bonds make important contributions to numerous fields of chemistry and biology. As examples, understanding the forces behind crystal engineering and supramolecular chemistry benefits from a knowledge of σ-hole interactions due to their directionality, strength, and self-organization properties which promote formation of adducts in the solid state [34,35,36,37,38,39,40,41,42,43,44,45,46,47,48,49,50,51,52]. The importance of σ-hole bonding has also been verified in the context of anion recognition processes [53,54,55,56,57,58,59,60,61], materials chemistry [62,63,64,65,66,67,68,69,70,71,72], or biochemistry [73,74,75,76,77,78,79,80,81]. An early work connecting σ-hole bonds with crucial concepts in chemistry occurred when Grabowski recognized that tetrel bonds can be thought of as a preliminary stage of the very important S_N_2 reaction [82].

**Figure 1:**
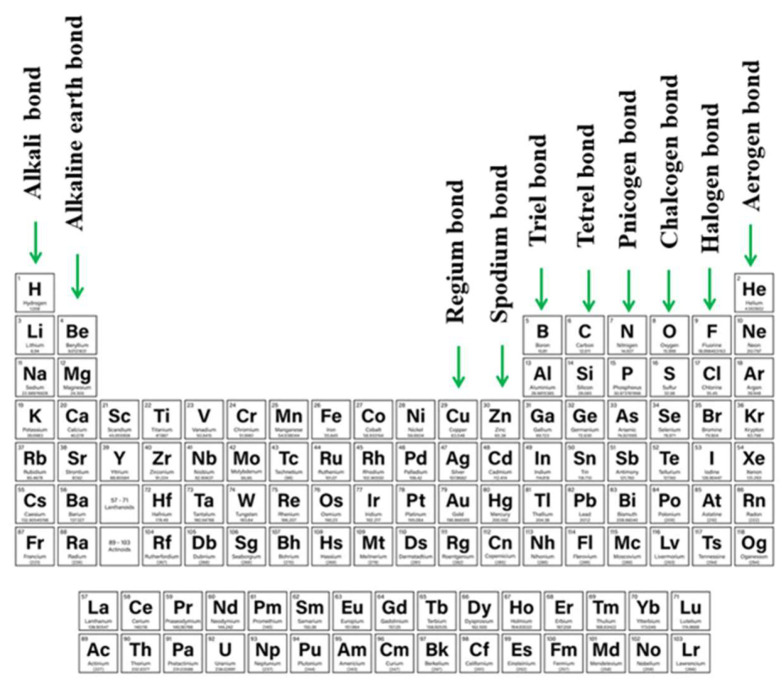
Family of σ and π-hole interactions.

As ideas concerning the σ-hole were proliferating, it was recognized that density depletion is not necessarily limited only to the extensions of covalent bonds. Depletions can also occur above planar groups as well, as for example above a carbonyl or phenyl group. Linear systems such as HCN can also suffer from low density off the molecular axis. Because of their location and association with π-electronic systems, these regions of density depletion and positive electrostatic potential have come to be called π-holes. As one specific example, tricoordinated triel atoms typically occur at the center of a planar triangle, with a π-hole located above the central triel atom [13,83,84,85,86,87], and the resulting triel bond [88,89,90,91,92,93,94,95] falls into the category of a π-hole bond. The π-hole situated above the C atom of a carbonyl group offers another common example, whose presence was manifested in early work of Burgi and Dunitz [96]. Protein structures can also involve participation of π-hole bonds [97,98], as is also true of self-assembling systems [99]. As work has progressed, there has been recognition of π-holes as providing a means by which an aerogen bond (involving rare gas atoms) can form [100,101,102,103,104]. Other newly introduced types of σ/π- hole directed interactions are alkali and alkaline earth bond (e.g., beryllium bond, magnesium bond) in which atoms of 1st and 2nd groups contribute [105,106,107,108,109] or regium and spodium bonds which employ transition metals from 11th (regium [110,111,112,113,114,115]) or 12th (spodium [105,116,117,118,119]) groups of the periodic table. The full range of these sorts of bonds, along with their designations, is summarized in Figure 1.

There have been a number of earlier reviews addressing the issue of σ-hole and π-hole bonds [13,49,51,52,81,84,105,120,121]. However, little attention has been devoted to situations where both hole types are present on a single molecule, and the competition between the two for a nucleophile. Indeed, it is also of intense interest to examine the result when both of these bonds are present at the same time. As has already been explored, the tunability of single σ or π-holes enables the construction of interesting assemblies with desired properties [122,123,124]. The possibilities multiply when both types of bonds are present and influence one another.

The driving goal for this work is to review with a critical eye what is known about systems offering both σ and π-holes to an approaching nucleophile. Are there rules that can be used to predict which will be preferred? Is there a distinction depending on whether both holes lie on the same atom or on different atoms of the same molecule? Different combinations are discussed where π-hole bonds of various types are combined with other noncovalent bonds, whether aerogen, halogen, tetrel, pnicogen, or chalcogen.

## 2. Origin of π-Holes

**Figure 2:**
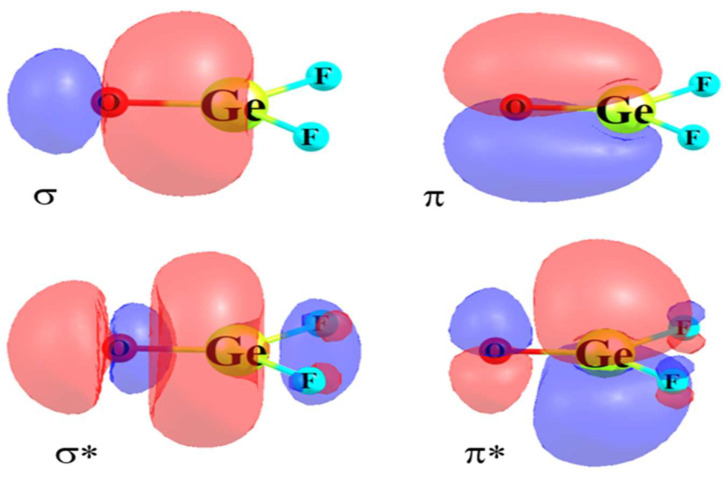
Ge-O NBO bonding and antibonding orbitals of GeF_2_O. Red and blue colors indicate opposite phase of the wave function.

**Figure 3:**
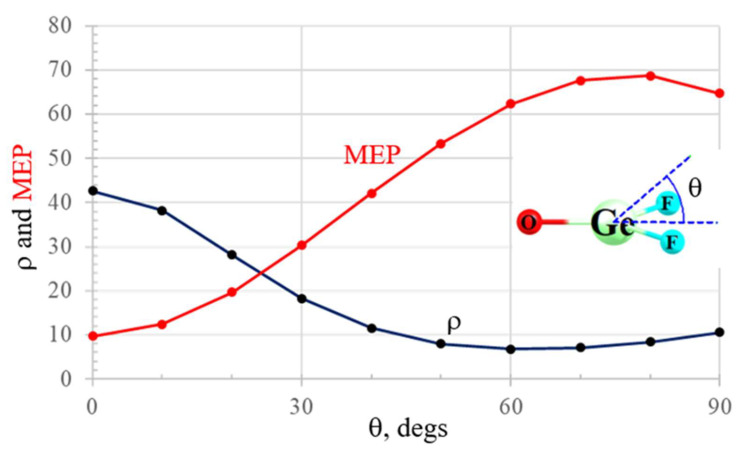
Electron density (10^−5^ au) and molecular electrostatic potential (MEP) (10^−3^ au) of GeF_2_O as a function of the displacement from the molecular plane θ, at a distance of 2.5 Å from the Ge atom.

**Figure 4:**
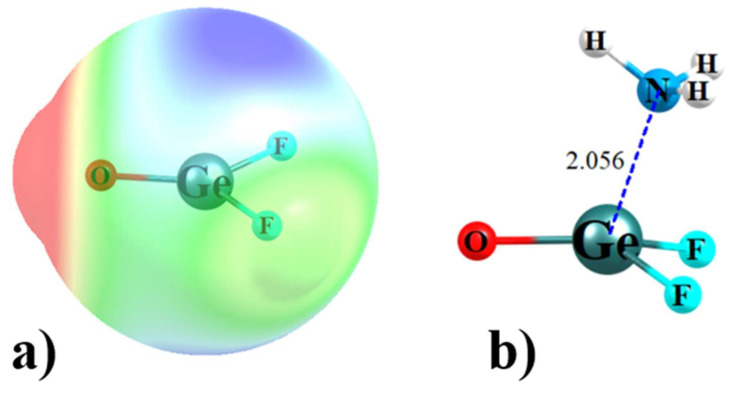
(**a**) MEP on surface of GeF_2_O defined as 1.5 × vdW atomic radii. Blue and red colors refer to +0.05 and −0.05 au, respectively. (**b**) Optimized complex of GeF_2_O with NH_3_ at the mp2/aug-cc-pVDZ level, with distance in Å.

The σ-hole has been well documented in the literature, along with its explanation as emanating from the pull of electron density along the axis of a covalent bond. The origin of a π-hole is similar in some ways, as it too relies on anisotropic distribution of charge. The fundamental origin and nature of a π-hole can be understood using F_2_GeO as an example. This molecule is planar with C_2v_ symmetry. The NBO localized orbitals representing the σ and π bonding orbitals of the Ge=O bond exhibited in the top half of Figure 2 both show a bias toward the more electronegative O. The region above the Ge atom and on its right, toward the two F atoms, thus suffers from a depletion of electron density. This shift is reflected by the drop in the total electron density as the point of reference moves up and out of the molecular plane, culminating in the minimum of the black ρ curve in Figure 3 for θ ~ 60°. Such a density depletion above the molecular plane is commonly referred to as a π-hole, which is in turn responsible for a maximum of the red molecular electrostatic potential (MEP) curve in Figure 3, which occurs at roughly θ = 75°. Figure 4a illustrates this π-hole in the MEP as the blue region lying above (and below) the Ge and shifted slightly toward the two F atoms. It is thus natural for a nucleophile to then approach this π-hole from this direction, as exemplified by the complex with NH_3_ displayed in Figure 4b. It might be noted as well that the π* orbital in Figure 2 is perfectly situated to act as electron acceptor from the lone pair of the NH_3_ nucleophile, another trademark of π-hole interactions. Note finally the geometrical distortion of the originally planar F_2_GeO in Figure 4b as the O and F atoms are shifted away from the approaching nucleophile another common characteristic of π-hole bonds.

Of course, the forgoing explanation of the origin of a π-hole can vary from one system to the next. The presence of any lone electron pairs on the central atom can influence the magnitude and actual location of any such π-hole. An example to be discussed shortly is XeF_2_O where the central Kr atom contains two such lone pairs. The positive region that appears above an aromatic ring of, e.g., C_6_F_6_, is not located directly above any one C atom but lies rather above the ring’s center. Such a dislocation can at times make it problematic to connect a π-hole with any particular atom, such as that above a C≡N group which lies roughly midway between the C and N atom.

It should be reiterated that the causality is as follows: depletion of electron density causes a rise in the electrostatic potential [125]. In fact, sometimes a hole is labeled as such even though the potential is not positive, just less negative than its surroundings. For practical reasons, the magnitude of a hole is typically measured on the 0.001 au electron isodensity surface and is quantified by the V_S,max_ parameter developed for this purpose [12,126]. The electron density can be accessed not only by quantum calculations but also experimentally by diffraction methods [127].

It has been shown that the intensity of a σ-hole can be adjusted by changing the polarizability of the central atom and the electron-withdrawing power of its substituents [13,84,127], and the same considerations apply to π-holes as well [128]. However, it must be borne in mind that the strength of a given interaction is not a function solely of electrostatic considerations. Polarization and dispersion forces are important attractive forces as well [13,17,129,130,131].

## 3. σ-Hole and π-Hole on the Same Atom

**Figure 5:**
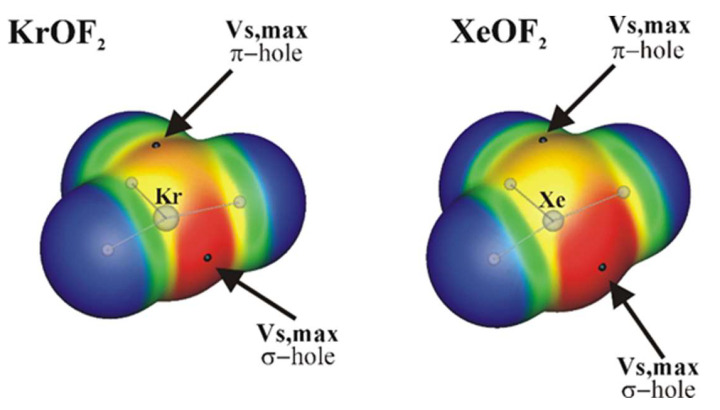
MEP of the isolated KrOF_2_ and XeOF_2_ molecules on the 0.001 au contour of the electron isodensity, at the MP2/aug-cc-pVDZ level. Color ranges, in kcal/mol, are red, greater than 40, yellow; between 20 and 40, green; between 0 and 20, blue, less than 0 (negative). Selected surface critical points V_s,max_ (σ and π-holes) are indicated as black dots. Reproduced from Reference [132] with permission from the PCCP Owner Societies.

There are only a few reports in the literature of systems where both σ and π holes appear on the same atom. One example of such a situation is furnished by AeF_2_O where Ae refers to an aerogen atom Kr or Xe [132]. As may be seen in Figure 5, the Ae atom of this molecule contains a σ-hole along the O=Ae bond extension, while a π-hole opens up above the molecular plane. As may be seen in the upper section of Table 1, the σ-hole is quite a bit more intense than the π-hole. The weaker nature of the latter may be attributed in part to the presence of lone pairs on the central Ae atom which share space with these holes and would dilute any density depletion. It is interesting to note that whereas the σ-hole is a bit more intense for Xe vs Kr, the reverse order is seen in their π-holes.

These two AeF_2_O molecules were allowed to react with a series of diazine nucleophiles (with negative MEP minima on the N atoms) [132]. It was found that the intensity of these various holes carries over into the interaction energies (E_int_) of the corresponding dimers. Complexation through the σ-hole was more stable than the corresponding π-hole dimers by about 6 kcal/mol. The stability order was parallel to the hole intensities, as the Xe complexes were slightly stronger than Kr atom analogues.

Another study by Bauza and Frontera [104] of related complexes obtained a deeper σ-hole on XeOF_2_ by a different computational procedure. XeOF_2_ complexes with ammonia and CH_3_CN were characterized by similar interaction energies as in Reference [132]. Interaction energies were magnified by the use of an anion as nucleophile as would be expected. Unfortunately, these authors did not consider π-hole complexes for purposes of comparison.

**Figure 6:**
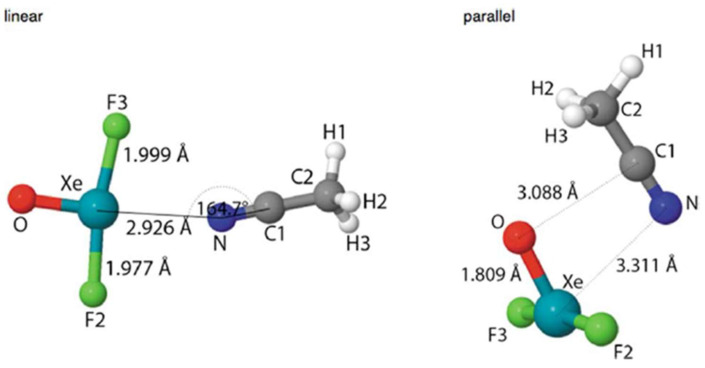
Two binding modes of XeOF_2_···NCCH_3_ complexes, from Reference [137].

The ability of XeOF_2_ to interact with a Lewis base through both hole types was considered [137] in connection with the nucleophile NCCH_3_, as displayed in Figure 6. The σ-hole complex on the left has the a shorter Xe···N distance than the π-hole structure by nearly 0.4 Å, and is preferred by 2–4 kcal/mol. This energetic advantage occurs despite the presence of a secondary C···O tetrel bond in the latter. The authors questioned the level of covalency in the Xe···N bond, but their topological analyses were not conclusive. Unfortunately, this work did not delve into the σ or π-hole depths as background information.

Brock et al. [138] provided experimental crystal structural evidence of the XeOF_2_···NCCH_3_ binding wherein three different nucleophiles approached Xe from three different directions simultaneously, two of π-hole character and one σ-hole, demonstrating the viability of multiple bonds to different holes of the same atom. In a different vein, the σ-hole at Xe atom in XeOF_2_ has also been a vehicle by which to illustrate cooperativity between regium and aerogen bond in the ternary systems of C_2_H_2_···MCN···XeOF_2_, C_2_H_4_···MCN···XeOF_2_, MCN···C_2_H_4_···XeOF_2_ and C_2_(CN)_4_···MCN···XeOF_2_ where M= Cu, Ag or Au [111]. The σ-hole was able to interact with a N lone pair as well as the unsaturated π- system of a C=C bond. It would have been particularly interesting had the authors considered similar questions with regard to the Xe π-hole.

Within a hypervalent bonding situation, halogen atoms are also capable of containing a π-hole. Within the context of the BrOF_2_^+^ cation [139] an X-ray structure indicated the central Br atom can be approached by three nucleophiles. Two KrF_2_ and one AsF_6_ molecule attack electrophilic regions on the outer surface of the Br atom. While these three Lewis bases appear to interact through σ-holes on the Br atom, it cannot be excluded that there is a π-hole site on this cation which can attract another Lewis base.

**Figure 7:**
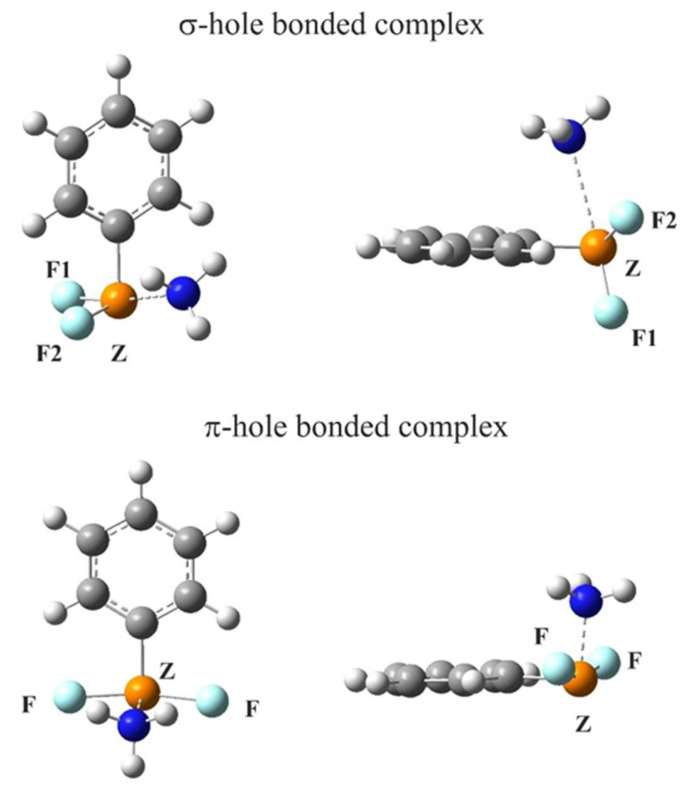
Two types of the MP2/aug-cc-pVDZ optimized structures (top and side views) of complexes of NH_3_ with ZF_2_C_6_H_5_, from Reference [133].

Turning to the pnicogen atom, interaction with NH_3_ [133] caused ZF_2_C_6_H_5_ (Z = P, As, Sb, Bi) to take on two different arrangements. The first contained three σ-holes in the range of 19-53 kcal/mol, the strongest for the most polarizable Bi atom. As indicated in Figure 7, approach to this σ-hole places the NH_3_ opposite one of the F atoms. Internal rearrangement of the ZF_2_C_6_H_5_ to a more planar structure opens up a π-hole above the Z atom. As delineated in Table 1, this hole has a magnitude between 36 and 61 kcal/mol, which can also attract the NH_3_ nucleophile, as depicted in the lower half of Figure 7. The greater depth of the π-holes leads to their larger interaction energies by a factor of 2 to 8. However, the internal rearrangement required to open up these π-holes is energetically costly, requiring between 16 and 43 kcal/mol, so that despite the greater depth of the π vs σ-holes, and their superior interaction energies, it is the set of structures in the top half of Figure 7 that are energetically preferred by a margin between 1 and 11 kcal/mol. This deformation energy diminishes with larger Z atoms, so that there is a closer competition between the σ and π-hole complex binding energies.

Another study of pnicogen bonded systems addressed the question of how many Lewis base ligands can be attached to a single ZF_3_ molecule [134]. The ZF_3_ monomer (Z = P, As, Sb, Bi) is characterized by three σ-holes, one opposite each Z-F bond, and a much shallower one that lies directly opposite the Z lone pair, amongst the three F atoms. Although ZF_3_ is pyramidal, the latter was considered a π-hole due to its placement. Table 1 documents the much lesser V_S,max_ of the latter as compared to the three σ-holes on each ZF_3_ unit. Because of its greater electrostatic attraction, it is the σ-hole that draws in the approaching nucleophile, whether HCN, CN^−^, or NH_3_. A second nucleophile of any sort occupies a second σ-hole, but such a triad is only possible for the two heavier Sb and Bi atoms for the anionic CN^−^ due to the large Coulombic repulsion required to form such a dianion, and even so, the SbF_3_··(CN^−^)_2_ triad has a positive binding energy. HCN is too weak a nucleophile to squeeze in a third pnicogen-bonded base, while such a tetrad is possible for NH_3_. Addition of a fourth NH_3_ is possible only for Z = Bi. It seems to occupy the π-hole mentioned above, but this weak interaction is reinforced by secondary noncovalent bonding, reflecting the reluctance of these units to engage with their weak π-holes.

**Figure 8:**
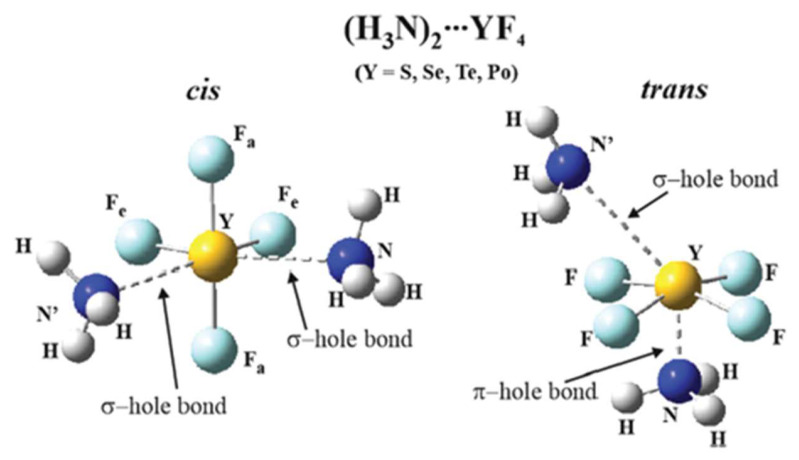
Cis and Trans arrangements of two NH_3_ units complexed with YF_4_ via chalcogen bonds. Reproduced from Reference [135] with permission from the PCCP Owner Societies.

Shifting attention to chalcogen bonding, the YF_4_ (Y = S, Se, Te, Po) monomer adopts a see-saw equilibrium geometry [135] with a pair of σ-holes lying opposite each of the two equatorial Y-F bonds, with V_S,max_ magnitudes between 42 and 76 kcal/mol, as listed in Table 1. A pair of NH_3_ nucleophiles can approach along these two σ-holes which would lead to an overall octahedral arrangement with the two NH_3_ units *cis* to one another, as depicted on the left side of Figure 8. An alternative trans positioning of the two nucleophiles as shown on the right side of Figure 8 would place the central YF_4_ in a square pyramidal configuration, closer to a square. As such, it would acquire a π-hole directly below the Y atom. It may be observed from Table 1 that these π-holes are rather intense, between 53 and 64 kcal/mol, so can easily attract one of the two NH_3_ bases. The other side of the nearly square YF_4_ unit contains a Y lone electron pair, which obstructs the second NH_3_ from approaching directly opposite the first, so it situates itself closer to a point opposite one of the Y-F bonds, close to its σ-hole. The energetic comparison of the *cis* and *trans* geometries must again consider both interaction and deformation energies. Whereas the interaction energies of the *trans* structure are much more negative than those for *cis*, the deformation energies required to adopt the nearly square structure are much larger as well. The net result is that the binding energies of the *cis* and *trans* conformations are comparable to one another. *Cis* is favored for the two smaller S and Se chalcogen atoms, while the heavier Te and Po, with their somewhat reduced deformation energy requirements, shift the equilibrium toward *trans*.

Similar considerations apply to tetrel bonds. TF_4_ (T = Si, Ge, Sn, Pb) is of course tetrahedral, so is characterized by four equivalent σ-holes opposite each T-F bond [136]. In order to accommodate a pair of bases, TF_4_ distorts into an octahedron. *Cis* approach of the two bases places the TF_4_ in a see-saw geometry with a pair of σ-holes, each lying opposite an equatorial T-F. An alternate deformation into a square planar structure, allowing for trans arrangement of the two bases, imparts a pair of π-holes to the TF_4_ unit. The lowermost section of Table 1 shows the σ-holes of the former structure are slightly deeper than the π-holes of the latter. It is worth stressing that either type of hole in these distorted arrangements, whether see-saw or square, is considerably deeper than those in the original undistorted tetrahedral geometry. The interaction energies of the pair of NCH bases with the square was considerably more negative than for the see-saw, despite the larger V_S,max_ for the latter, by between 10 and 40 kcal/mol. On the other hand, the energy required to deform the tetrahedral TF_4_ into a square far exceeded that needed for the see-saw, particularly for the smaller T atoms. In quantitative terms, the deformation energies for the squares varied from 22 all the way up to 66 kcal/mol for SiF_4_, as compared to only 0.4–15 kcal/mol for the see-saw. The net result is that it is the *cis* geometry that is favored, and this preference varies from only 3 kcal/mol for PbF_4_ up to 23 kcal/mol for SiF_4_. In fact, the large deformation energies for the square structures with small T make the binding energy a positive quantity for both SiF_4_ and GeF_4_.

The forgoing highlights the importance of considering π-holes, even for Lewis acids whose undistorted monomer geometries are such that no such holes are present. The deformations which the monomer undergoes in its interaction with one or more bases can present the possibility of a π-hole whose interaction is comparable to, or even stronger than, that with the original σ-holes.

## 4. σ-Hole and π-Hole on Different Atoms 

More common than the presence of a σ and π-hole arising on the same atom is the situation wherein these two holes are located on different atoms. Within this subgroup there are several themes that are more common than others.

### 4.1. Combination of σ-Hole on Halogen or Chalcogen with π-hole on Aromatic Ring

A number of works have considered situations wherein a σ-hole on a halogen (X) atom coexists with a π-hole lying above the plane of an aromatic ring. When not directly above the center of the ring, the π-hole is shifted so as to lie closer to the midpoint of a C-C or C-N bond, a topic which has been discussed at some length elsewhere [140]. With this understanding, this review subsumes all of these types into the category of π-hole. Values of the MEP positive maxima are enumerated in Table 2.

One prominent example modeled functionalization of graphene sheets by C_6_H_5_Br [141] and its physisorption on the graphene surface through either its Br σ-hole or phenyl π-hole. The adsorption energy with electron-rich graphene regions was three times larger for the π-hole interaction than for the σ-hole contact. Taking the analysis one step further, the authors noted that the adsorption seriously affects the properties of graphene. Bromopentafluorobenzene also has the option of interaction through either its σ or π-hole [142], in this case with pyridine. The latter nucleophile was able to interact either through its N lone pair or the π-electron system of its aromatic ring. As in the previous case the π-hole···π interaction yielded the more stable dimer, here by about 4 kcal/mol. As a fundamental point of distinction, energy decomposition suggested that whereas electrostatics is the dominant factor in σ-hole complexation, this role is assumed by dispersion in π-hole bonding. Another study of this type [143] involved homo-dimerization of 1,3,5-trifluoro-2,4,6-triiodobenzene (TITFB) through its σ or π holes. The σ-hole on the iodine atom was roughly three times deeper than the π-hole positioned above the benzene ring. However, the intermolecular distance was shorter of about 0.1 Å in the case of π-hole interaction.

**Table 2:** Comparison between σ and π holes appearing on the same molecule, where σ-hole is coupled with halogen (or chalcogen) atom and π-hole with an aromatic ring, V_S,max_ in kcal/mol.

Molecule	Hole Source	Hole Type	V_S,max_	References
1,4-DITFB	I	σ	32.3	[140]
	Carbon/Benzene ring	π	15.1	[140]
C_6_H_5_Br	Br	σ	up to ~15	[142]
	Benzene Ring	π	up to ~15	[142]
TITFB	I	σ	30.1	[143]
	Carbon/Benzene Ring	π	11.4	[143]
Halogen Substituted 1,3,4-oxadiazol-2Ĳ3H)-thiones	S (Chalcogen), Cl, Br	σ	up to ~37	[144]
	Oxadiazole and Benzyl Ring	π	up to ~37	[144]
Haloperfluorobenzene	Cl, Br, I	σ	20.9 to 32.8	[145]
	Benzene Ring	π	12.6 to 19.8	[145]
XC_3_H_4_N_2_^+^; X = F, Cl, Br, I	Cl, Br, I	σ	105.9 to 117.5	[146]
	Imidazolium Ring	π	111.0 to 127.9	[146]

Shukla et al. [144] found that biologically active derivatives of halogen substituted 1,3,4-oxadiazol-2Ĳ3H)-thiones in the solid state contain a σ-hole on the S, Cl, and Br atoms and a π-hole above the oxadiazole and benzyl rings all of which participate in noncovalent interactions. The S···N σ-hole chalcogen bond manifested distinctive activity in stabilization of these amalgams. This work fits into the idea that both σ and π-hole bonding play a pivotal role in the 3D organization of crystalline structures and in various molecular scaffolds.

Li et al. provided another example [145] in the context of interactions between haloperfluorobenzene with fluoroanthene (FA) which assemble into nine luminescent cocrystals. These interactions involved the participation of the σ-hole of Cl, Br, or I atoms or the π-hole of the phenyl ring with aromatic π-electrons on the FA. The σ-holes were more intense than the π-hole by as much as 17 kcal/mol. While the balance between σ and π-hole bonding with FA varied as the σ-hole deepened and the π-hole weakened along with the growth of the halogen atom, it was nonetheless the σ-hole···π interaction that was universally favored.

Wang et al. [146] examined the interaction between protonated 2-halogenated imidazolium cation (XC_3_H_4_N_2_^+^; X = F, Cl, Br, I) and the set of (CH_3_)_3_SiY (Y = F, Cl, Br, I) Lewis bases. The acid contained both a σ-hole on its X atom and a π-hole above the imidazolium ring. The π-holes were the deeper of the two, leading to stronger interactions. In these ionic cases, both interaction types were driven mainly by electrostatic forces (the electrostatic term was well correlated with the V_S,max_ values found on monomers) with some addition of polarization and dispersion. The dispersion term was somewhat more prominent for the weaker σ-hole complexes while the polarization component towered over dispersion for π-hole dimers.

An experimental component was contributed by Zhang et al. [147], which paired C_6_F_5_X (X = Cl, Br, I) with C_6_D_6_ in solution. As in the earlier cases, a σ-hole appeared on the X atom while a π-hole occupied the space above the aromatic ring. For the strongest σ-hole which was associated with the I atom, the interaction lay through its σ-hole, while it was the π-hole link that dominated for the smaller halogens. This pattern was attributed by the authors in large part to entropic contribution since enthalpy alone would not be sufficient to stabilize σ-π over π-π. Comparable conclusions were drawn in another paper by the same group [148], where Lewis acids participated in complexes with deuterated solvent molecules with lone-pair electrons, including CD_3_CN, CD_3_COCD_3_, CD_3_OD, and [D_6_]DMSO. Experiment and calculations showed that again σ-hole and π-hole bonds compete with each other for the nucleophile’s lone pair, rather than the π-electron system of C_6_D_6_ in the earlier work. The results indicated that only the iodine and a few bromine σ-hole halogen bonds are strong enough to contend successfully with the π-hole interactions in solution.

The biologically relevant ebselen derivative, 2-(2-bromophenyl)benzo[d][1,2]selenazol-3(2H)-one homodimer [149] exhibited markers of simultaneous σ-hole and π-hole bonding. The σ-hole lay along the C-Br covalent bond elongation and the π-hole was derived from the phenyl ring. Shukla et al. postulated two simultaneous interactions: σ-hole bonding from the Br σ-hole to the negative charge-concentrated C-C bond, as well as a π-hole bond between a positively charged C atom and the lone electron pair of Br, confirmed by their NBO analysis. This case represents an uncommon model wherein the same atom acts as both σ-hole bond acceptor and π-hole bond donor.

Finally, Wang et al. [140] reviewed a comparison between complexes stabilized by σ/π holes with various nucleophilic acceptors with particular emphasis on their potential application to anion recognition and transport. Among numerous examples, one of especial interest concerned 1,4-diiodoperfluorobenzene (1,4-DITFB) with both a σ and π-hole. The former was twice as deep as the latter, and the authors underscored that this molecule can act as σ/π hole donor and also by taking into account the high amount of electronic density collected on F atoms, and on the belt around the central fragments of the I atoms surface, it can serve as σ-hole or π-hole acceptor as well.

### 4.2. Combination of σ-Hole Halogen Bond with Other π-Hole Sources

Further exploration of the literature supplies examples where a X σ-hole is combined with π-holes derived from groups other than aromatic rings. Instances of the halogen-pnicogen combination are most numerous. Lang et al. [150] provided a textbook example in that the surface of XONO/XONO_2_ (X = F, Cl, Br, I) contains both a typical σ-hole on X and a π-hole localized on the N atom. The V_S,max_ values for this set of systems are listed in the first four rows of Table 3. The authors observed that the σ-hole intensity grew in line with the increase of X atom size whereas the π-hole magnitude dropped in this same order (similar picture as at Li et al. [145]). Binary complexes of these monomers with ClO and ammonia had interaction energies correlated to the MEP maxima. The ternary ClO···XONO/XONO_2_···NH_3_ complexes revealed anti-cooperative effect between the σ-hole halogen and π-hole pnicogen bond, as expected when the central unit acts as double electron acceptor. Solimannejad et al. [151] examined adducts of NO_2_X (X = Cl, Br) with HCN and HNC moieties. The π-hole was stronger than the σ-hole, contrary to the Lang et al. results. Within four tested interaction schemes in binary complexes (stabilized by σ-hole, π-hole and two different hydrogen bond approaches), those bonded through the π-hole were the most stable, for the majority of the trimers displayed negative cooperativity between the σ and π-hole interactions. Subsequent work of this research group [152] deployed the NO_2_I monomer with a different Lewis base (ammonia). The I atom showed a deeper σ than π-hole, which led to the expected outcome that the NO_2_I dimer with ammonia was more strongly bound by its σ than by its π-hole. This study also supported two conclusions formulated in earlier cited works: (i) both interaction types showed a strong correlation between V_S,max_ and interaction energy, and (ii) antagonistic effect between σ and π-hole bonds was observed in trimers with two ammonia units.

**Figure 9:**
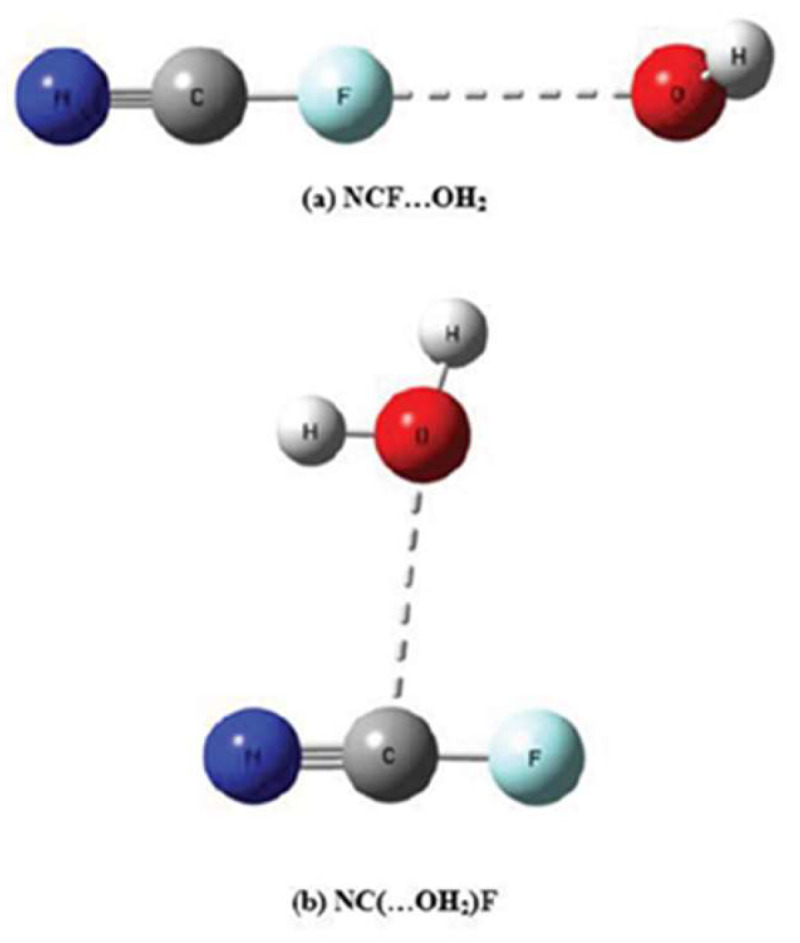
Optimized structures of NCF complexes with water stabilized by (**a**) σ-hole and (**b**) π-hole, from Reference [153].

There are also examples in the literature combining a σ-halogen with a π-tetrel bond. As one example, McDowell [153], analyzed complexes of NCX (X = F, Cl, Br) with H_2_O. Besides the obvious appearance of a σ-hole on X, a π-hole region was noticed above and below C. As in the previous cases, the intensity of the π-hole drops along with larger X as its σ-hole deepens. Of the two potential binding modes illustrated in Figure 9, only NCF was able to form complexes with water via both o modes a and b. Whereas the π-hole on NCF was 2.5 times stronger than its σ-hole, the π-holes on NCCl and NCBr were too weak to attract the incoming nucleophile. With regard to addition of a third entity, addition of hydrogen and beryllium cations destabilized the σ-hole bond while improving binding in the π-hole complexes.

Bauza and Frontera [154] tested the ability of BH_2_X (X = F, Cl, Br, I) to establish various sorts of interactions in their homodimers, depending on the attack angle between them. Along with the presence of a σ-hole on X, a π-hole was also available over the central B, perpendicular to the molecular plane. The strengths of these two holes followed expected trends based on X atomic size, and the B π-hole was considerably more intense than the X σ-hole. It was therefore no surprise to find a strong interaction energy of −34.7 kcal/mol for π-hole(B)···σ-hole(I) interaction, much larger than that when I is replaced by Br, or even the halogen bond between I σ-holes in a pair of BH_2_I molecules.

A particularly unusual combination of holes was examined by Alikhani [109]. The π-hole on BeCl_2_ occurs as a narrow belt around the Be atom, with V_S,max_ = 32.2 kcal/mol. The magnitude of the σ-hole on Cl is only 1.2 kcal/mol, too weak to expect effective attraction of a Lewis base. The author consequently focused on the nature and properties of the π-hole beryllium bond in complexes with nucleophiles Cl_2_, NH_3_, and DMF (dimethylformamide), all of which proved to be quite strong, with the E_int_ reaching up to −46.3 kcal/mol. One might expect that the replacement of the Cl on BeCl_2_ by Br or I would intensify the X σ-hole and perhaps also weaken the π-hole on Be. If that were the case, then a XB through the Br or I σ-hole might be able to successfully compete with the Be π-hole interaction.

### 4.3. Other Examples

The literature contains a few other sorts of combinations of σ with π-holes. Pal et al. [155] present the interplay between these binding sites in Fmoc-Leu-Ψ[CH_2_NCS] (Fmoc = fluorenylmethyloxycarbonyl protecting group, Leu = leucine) organic isothiocyanate which act as an intermediate in the synthesis of bioactive peptides. The N=C=S group contains a σ-hole on the S atom surface (along the extension of the C=S bond), and a π-hole perpendicular to the C-N bond. The specific location of the π-hole makes it difficult to classify as either a π-hole tetrel or pnicogen bond. For the purpose of the current review, it was classified as π-hole tetrel/pnicogen bond (see Table 4). Between these positively charged sections, there were also regions of negative potential derived from the lone electron pair of the nitrogen (σ-hole acceptor) and sulphur (π-hole acceptor) atoms. N=C=S···N=C=S contacts were observed in the crystal lattice that are associated with electrostatically driven attractions between regions of opposite charge. These simultaneous σ and π-holes interactions with the negative sites were cooperative, thereby magnifying the stabilization within the crystal.

The specific location of the π-hole was more definitive [156] in CF_2_=CFZH_2_ (Z = P, As, Sb) and CF_2_=CFPF_2_. The electron-deficient regions corresponding to π-holes were positioned directly above the C atom, while the σ-hole was localized on the pnicogen Z atom. The σ-hole became deeper in the sequence P < As < Sb attributed to rising Z polarizability, while the π-hole magnitude lessens in the same order, as documented in Table 4. Switching -PH_2_ moiety to -PF_2_ boosted the magnitude of both holes. The prediction of MEP extrema was verified by the energetic properties of the complexes formed between these Lewis acids and NH_3_ or NMe_3_. The interaction energies of the dyads were generally consistent with the MEP trends. However, some irregularity was found, as the σ-hole complexes in each case were more stable than their π-hole counterparts, as might be expected for Z = As, Sb but counter to MEP trends for P. It is worth emphasizing, however, that the differences in energy between the two kinds of complexes were less than 0.5 kcal/mol. It might be concluded then that σ-hole and π-hole sites can compete with one another in these systems. Further analyses (NBO, E_int_ decomposition) revealed that the formation of the complexes is grounded not only in the electrostatic term (which nevertheless governs) but also charge transfer between subunits which varied from 0.3 up to 14.4 kcal/mol, for LP(N) → σ*(C-Sb) donation. The scale of charge transfer was larger for σ-hole dimers than in the case of π-hole adducts.

Another work by this group [157] involved the F_2_C=CFTF_3_ (T = C, Si, and Ge) as Lewis acid with water, ammonia, or formaldehyde as Lewis base, comparing the σ or π-holes on the tetrel T atoms. The σ-hole was formed along the T-F bond while the π-hole appeared perpendicular to the C=C double bond, coplanar to the σ-hole. The π-hole maxima rose along with the T atom size, in contrast to the case in earlier studies. However, the magnitudes were quite limited and for T = C and Si, the π-hole intensities were very close to one another. One can assume that the π-hole connected with the C=C bond is much less sensitive to the remainder of the molecule. The MEPs of the σ-holes were mostly consistent with previous patterns and also increased with the enlargement of T. The π-hole was stronger than the σ-hole in F_2_C=CFCF_3_ while the reverse was observed for T = Si and Ge. In keeping with this MEP data, F_2_C=CFCF_3_ prefers to form a π-hole tetrel bond whereas F_2_C=CFSiF_3_ and F_2_C=CFGeF_3_ were prone to a σ-hole tetrel bond. The strong correlation between MEP and interaction energy of the dimers was confirmed.

## 5. Conclusions and Prospective

Molecular systems can contain both σ- and π-holes, and there is a growing literature on both of them that allow some comparisons to be drawn. The presence of both sorts of holes is reflected in an expansion of possible binding sites and an increased flexibility in the noncovalent bonding with nucleophiles. The examination of crystals displays this flexibility in an array of binding patterns [104,140,141,142,143,144,145,146,149,153,155] where the directionality of σ/π-hole bonds is integral to arrangement, stabilization, and self-organization. There are numerous examples where these interactions are important to systems with biological connections [144,149,155] or photophysical performance [141,143,145].

In addition to the crystallographic data and their supporting theoretical investigations, examinations of systems in solution or model geometries in the gas phase provide additional insights into mutual presence of both σ and π-holes. The most-commonly observed situation to date is the combination of a σ-hole on a halogen atom with a π-hole from a variety of sources, most notably an aromatic ring. However, the full list is rather extensive, as for example when both sorts of hole occur on a single tetrel atom. One interesting conclusion is that a particular sort of hole does not have to be present in the isolated monomer. For example, the approach of a nucleophile can induce geometric deformation into the Lewis acid which in turn causes the appearance of a π-hole that is not present in its isolated form.

Another pattern which has emerged from the studies is that a σ-hole typically deepens as the atom on which it occurs grows larger, e.g., Si < Ge < Sn, whereas this same enlarging atom can lead to a weakening of a π-hole. These opposing trends offer the opportunity to guide an emerging complex geometry based on atom size. When both hole types are present, they each offer an attractive site for binding by a nucleophile. However, since both bonding types utilize the Lewis acid as electron acceptor, the two bonds weaken one another in a negative cooperative manner.

Whether σ or π-hole type, the noncovalent bond appears to have a strong electrostatic component, based on rigorous calculations of this component, as well as a tight correlation of bond strength with the depth of the hole. However, these bonds rely to a large extent also on polarization and charge transfer effects, complemented by dispersive forces, although the precise mix of these different components varies from one bond to another. A slightly different perspective on some of these bonds has been offered by the Bickelhaupt group [22,23] in which emphasis is placed on the activation strain model of chemical reactivity and the energy decomposition analysis combined with molecular orbital theory.

It is hoped that this review of the current state of knowledge concerning these σ and π-hole bonds will motivate additional work to better refine our understanding of the forces that undergird and control them. In a practical sense, this enhanced knowledge base will hopefully lead to the development of new crystal packing motifs and to innovative materials with improved properties.

## Figures and Tables

**Table 1:** Comparison between σ and π hole depths in molecules where they coexist on the same atom, V_S,max_ in kcal/mol.

Molecule	Hole Source	Hole Type	V_S,max_	References
Aerogen Bond Donors				
KrOF_2_	Kr	σ	58.7	[132]
KrOF_2_	Kr	π	39.1	[132]
XeOF_2_	Xe	σ	63.4	[132]
XeOF_2_	Xe	σ	90	[104]
XeOF_2_	Xe	π	36.2	[132]
Pnicogen Bond Donors				
PF_2_C_6_H_5_	P	σ	19.4	[133]
PF_2_C_6_H_5_	P	π	36.2	*
AsF_2_C_6_H_5_	As	σ	28.2	[133]
AsF_2_C_6_H_5_	As	π	44.0	*
SbF_2_C_6_H_5_	Sb	σ	38.4	[133]
SbF_2_C_6_H_5_	Sb	π	56.6	*
BiF_2_C_6_H_5_	Bi	σ	52.6	[133]
BiF_2_C_6_H_5_	Bi	π	60.9	*
PF_3_	P	σ	35.6	[134]
PF_3_	P	π	9.7	[134]
AsF_3_	As	σ	43.9	[134]
AsF_3_	As	π	7.1	[134]
SbF_3_	Sb	σ	51.6	[134]
SbF_3_	Sb	π	10.6	[134]
BiF_3_	Bi	σ	61.5	[134]
BiF_3_	Bi	π	12.7	[134]
Chalcogen Bond Donors				
SF_4_	S	σ	41.7	[135]
SF_4_	S	π	64.4	[135] **
SeF_4_	Se	σ	51.2	[135]
SeF_4_	Se	π	61.1	[135] **
TeF_4_	Te	σ	59.2	[135]
TeF_4_	Te	π	54.5	[135] **
PoF_4_	Po	σ	76.3	[135]
PoF_4_	Po	π	53.2	[135] **
Tetrel Bond Donors				
SiF_4_	Si	σ	127.3	[136]
SiF_4_	Si	π	109.8	[136]
GeF_4_	Ge	σ	120.9	[136]
GeF_4_	Ge	π	106.1	[136]
SnF_4_	Sn	σ	129.4	[136]
SnF_4_	Sn	π	121.3	[136]
PbF_4_	Pb	σ	127.3	[136]
PbF_4_	Pb	π	104.1	[136]

* Value obtained by additional calculations for the purpose of the current review, at the same level as in Reference [136]. ** Values obtained for monomer in deformed complex geometry.

**Table 3:** Comparison between σ and π holes appearing on the same molecule, where σ-hole is coupled with halogen atom and π-hole with different sources (other than aromatic ring), V_S,max_ in kcal/mol.

Molecule	Hole Source	Hole Type	V_S,max_	References
Halogen-Pnicogen				
XONO (X = F, Cl, Br, I)	X	σ	−6.3 to 51.1	[150]
XONO (X = F, Cl, Br, I)	N	π	20.8 to 29.5	[150]
XONO_2_ (X = F, Cl, Br, I)	X	σ	2.7 to 67.7	[150]
XONO_2_ (X = F, Cl, Br, I)	N	π	29.6 to 41.2	[150]
NO_2_X (X = Cl, Br)	X	σ	13.2 and 19.0	[151,152]
NO_2_X (X = Cl, Br)	N	π	28.1 and 29.8	[151,152]
NO_2_I	I	σ	29.4	[152]
NO_2_I	N	π	23.5	[152]
Halogen-Tetrel				
NCX (X = F, Cl, Br)	X	σ	14.3 to 42.1	[153]
NCX (X = F, Cl, Br)	C	π	12.4 to 27.3	[153]
Halogen-Triel				
BH_2_X (X = F, Cl, Br, I)	Cl, Br, I	σ	3.5 to 11.3	[154]
BH_2_X (X = F, Cl, Br, I)	B	π	28.8 to 39.4	[154]
Halogen-Beryllium				
BeCl_2_	Cl	σ	1.2	[109]
BeCl_2_	Be	π	32.2	[109]

**Table 4:** Comparison between other combinations of σ and π holes appearing on the same molecule, V_S,max_ in kcal/mol.

Molecule	Hole Source	Hole Type	V_S,max_	References
Chalcogen-Tetrel/Pnicogen				
Fmoc-Leu-Ψ[CH_2_NCS]	S	σ	3.9 (exp.), 7.8 (theory)	[155]
Fmoc-Leu-Ψ[CH_2_NCS]	C=N bond	π	no data	[155]
Pnicogen-Tetrel				
CF_2_=CFZH_2_ (Z = P, As, Sb)	Z	σ	19.4 to 28.9	[156]
CF_2_=CFPF_2_	C	π	36.4	[156]
CF_2_=CFZH_2_ (Z = P, As, Sb)	Z	σ	25.7 to 28.2	[156]
CF_2_=CFPF_2_	C	π	40.1	[156]
Tetrel-Tetrel				
F_2_C=CFTF_3_ (T = C, Si, Ge)	T	σ	8.2 to 46.4 *	[157]
F_2_C=CFTF_3_ (T = C, Si, Ge)	C=C Bond	π	30.7 to 35.1 *	[157]

* In cited work, these values are probably given in wrong unit (eV instead of more reliable au).

## Data Availability

No new data were created or analyzed in this study. Data sharing is not applicable to this article.
